# A Revised Spiralian Homeobox Gene Classification Incorporating New Polychaete Transcriptomes Reveals a Diverse TALE Class and a Divergent Hox Gene

**DOI:** 10.1093/gbe/evy144

**Published:** 2018-07-07

**Authors:** Thomas B Barton-Owen, Réka Szabó, Ildiko M L Somorjai, David E K Ferrier

**Affiliations:** 1 Gatty Marine Laboratory, The Scottish Oceans Institute, School of Biology, University of St. Andrews, United Kingdom; 2 The Biomedical Sciences Research Complex, School of Biology, University of St. Andrews, United Kingdom

**Keywords:** *Spirobranchus lamarcki*, regeneration, operculum, biomineralization, NK genes, PRD class genes

## Abstract

The diversity of mechanisms and capacity for regeneration across the Metazoa present an intriguing challenge in evolutionary biology, impacting on the burgeoning field of regenerative medicine. Broad taxonomic sampling is essential to improve our understanding of regeneration, and studies outside of the traditional model organisms have proved extremely informative. Within the historically understudied Spiralia, the Annelida have an impressive variety of tractable regenerative systems. The biomeralizing, blastema-less regeneration of the head appendage (operculum) of the serpulid polychaete keelworm *Spirobranchus* (formerly *Pomatoceros*) *lamarcki* is one such system. To profile potential regulatory mechanisms, we classified the homeobox gene content of opercular regeneration transcriptomes. As a result of retrieving several difficult-to-classify homeobox sequences, we performed an extensive search and phylogenetic analysis of the TALE and PRD-class homeobox gene content of a broad selection of lophotrochozoan genomes. These analyses contribute to our increasing understanding of the diversity, taxonomic extent, rapid evolution, and radical flexibility of these recently discovered homeobox gene radiations. Our expansion and integration of previous nomenclature systems helps to clarify their cryptic orthology. We also describe an unusual divergent *S. lamarcki Antp* gene, a previously unclassified lophotrochozoan orphan gene family (*Lopx*), and a number of novel *Nk* class orphan genes. The expression and potential involvement of many of these lineage- and clade-restricted homeobox genes in *S. lamarcki* operculum regeneration provides an example of diversity in regenerative mechanisms, as well as significantly improving our understanding of homeobox gene evolution.

## Introduction

The capacity to regenerate missing tissues is widespread across the Metazoa, but the mechanisms by which it is achieved vary substantially between even closely related taxa, and much remains to be understood about the molecular bases of these processes. In 1901, T.H. Morgan proposed what has proven to be a resilient distinction between epimorphic regeneration, in which the replacement tissue is produced via cellular proliferation, and morphallactic regeneration, in which the tissue proximal to the wound is remodeled into a smaller version of the complete body part without proliferation at the wound site (Morgan 1901). Despite the breadth of taxon sampling that informed Morgan’s understanding of regeneration (Sunderland 2010), the categorization has not always been found to hold strictly true; many species that engage in epimorphosis also engage either simultaneously or sequentially in morphallactic remodeling (Özpolat and Bely 2016), whereas other regenerative mechanisms defy categorization when examined with modern tools. There are also substantial differences in the cellular mechanisms underlying examples of each type of regeneration (e.g., the wide variety of replacement tissue origins, [Tiozzo and Copley 2015]).

In response, some authors have called the usefulness of the nomenclature into question. Agata et al*.* (2007) hypothesized that all regeneration can be understood as a process of distalization, in which the distal-most portion of remaining (or new, undifferentiated) tissue is given the identity of the distal-most portion of lost tissue, followed by intercalation, in which the incongruous juxtaposition of identities causes the growth of intermediate tissues. However, Roensch et al.’s (2013) analysis of the expression of HOXA proteins in salamander limb regeneration indicated that this system uses an embryogenesis-like proximal-to-distal specification pattern, refuting the universal distalization/intercalation model that had otherwise gained broad support.

Homeobox genes are a transcription factor superclass defined by the presence of the homeodomain, a highly conserved helix-turn-helix DNA binding domain typically 60–63 amino acids in length. Precise spatiotemporal control of homeobox gene expression is used to orchestrate an enormous variety of vital aspects of development, and these roles are often ancient and deeply conserved. Among the most renowned of these is the determination of axial position (Hrycaj and Wellik 2016). Homeobox genes also hold an important position in our understanding of regeneration because they offer a convenient and robust way of understanding the control processes underlying regeneration and comparing them with the developmental ontogenesis of the same structures (see Roensch et al. [2013], as an important example). Widespread involvement of homeobox genes has been reported in diverse models of regeneration (Gardiner and Bryant 1996; Stierwald et al. 2004; Gersch et al. 2005; Alvarado and Tsonis 2006; Somorjai et al. 2012; Ben Khadra et al. 2014).

Annelids are important and very diverse models of regeneration (Bely 2006; Zattara and Bely 2011; Ferrier 2012; Balavoine 2014; Bely 2014; Kostyuchenko et al. 2016; Özpolat and Bely 2016; Boilly et al. 2017), and are proving extremely beneficial for bilaterian-wide comparisons of a number of biological processes (Christodoulou et al. 2010; Dray et al. 2010; Tomer et al. 2010; Simakov et al. 2013; Boyle et al. 2014; Lauri et al. 2014), in part because annelid genomes have generally evolved conservatively relative to other, perhaps more traditional, invertebrate model species (Raible et al. 2005; Hui et al. 2009, 2012; Ferrier 2012). Recent studies of annelid regeneration focus almost exclusively on antero-posterior segmental regeneration, which follows a stereotyped morphological sequence of wound healing, blastema formation, blastema patterning, differentiation, and growth (Bely 2014; Özpolat and Bely 2016). However, there are clear differences, notably with regards to the presence/absence of morphallactic processes occurring proximally to the dissection plane, even between closely related species (Licciano et al. 2012).

Analyses of homeobox gene expression in annelid regeneration have so far been limited to the Hox (Pfeifer et al. 2012; Novikova et al. 2013; de Jong and Seaver 2016) and ParaHox genes (Kulakova et al. 2008) in nereids and *Capitella teleta*. Hox expression in the regenerative blastema seems to be ancestral to the annelids (Özpolat and Bely 2016). They do not exhibit spatial or temporal collinearity of regenerative expression, indicating that they are not recapitulating embryogenic roles. Consistent with evidence on the diversity of regeneration mechanisms in annelids (Licciano et al. 2012), differences are observed in extent of proximal morphallaxis; *Alitta virens* undertakes substantial Hox expression reconfiguration (Novikova et al. 2013), whereas *C. teleta* exhibits relatively little change (de Jong and Seaver 2016).

*Spirobranchus* (formerly *Pomatoceros*) *lamarcki* is a serpulid worm that builds calcareous habitation tubes on the hard substrata in the marine environment of Northern Europe. The operculum, an evolutionarily novel head appendage (Bok et al. 2017), is used to plug the mouth of this tube, and contains muscular, vascular, and nervous tissue as well as a calcareous distal plate. *S. lamarcki* can completely regrow the operculum over the course of about 2 weeks after removal by dissection or its own autotomic response to attack. The regenerative process is comprised of the proliferation-less morphallactic remodeling of the tissue underlying the wound into the distal cup and plate region of the operculum, and the growth of the opercular filament from the intermediate tissue (Bubel and Thorp 1985; Szabó and Ferrier 2014). This process differs from stereotypical annelid caudal regeneration in lacking a blastema and in having a distal, rather than proximal, morphallactic component. *S. lamarcki* is distinctive amongst annelid model systems for its regeneration of a nonsegmental, histologically diverse, evolutionarily novel, biomineralizing appendage.

Much of the research on homeobox genes has focused primarily on genes belonging to families that were present in the genome of the ancestor of all bilaterally symmetrical animals. These orthology groups are well-conserved in modern genomes and, even though they frequently undergo gene duplication, it is usually possible to determine their orthology to these bilaterian families, often using only the sequence of the homeodomain. Taxonomically restricted, difficult-to-classify homeobox genes have been widely described, but are usually relatively modest in numbers and distribution, and the classification, evolution, expression, and function of these genes often goes ignored. Recent lophotrochozoan genome-wide homeobox surveys (Paps et al. 2015; Zwarycz et al. 2016) have revealed substantially greater numbers of these cryptic homeoboxes than in ecdysozoan or deuterostome genomes.

Paps et al. (2015) found that 31 of the 136 homeobox genes in the genome of *Crassostrea gigas* could not be assigned to ancient families, though the majority of these could be assigned to a class within the homeobox superclass, particularly the TALE and PRD classes. They concluded on the basis of homeodomain sequence phylogenies including a taxonomically broad sampling of difficult-to-classify homeobox genes that it was possible to assign these sequences to 19 clades, approximately but not definitely corresponding to taxonomically restricted orthology groups within the Spiralia (referred to by Paps et al. as Lophotrochozoa, sensu lato. Lophotrochozoa is used herein sensu stricto; c.f. Luo et al. 2018). Morino et al. (2017) examined a partially overlapping data set of spiralian TALE class sequences. They concluded that the majority of these sequences are monophyletic, presumably deriving from a single basal TALE homologue. However, a reconciliation of the Paps et al. (2015) and Morino et al. (2017) data sets and nomenclatures has not yet been attempted.

We present a survey of the homeobox-containing gene content of transcriptomes produced from different stages of *S. lamarcki* operculum regeneration. To aid classification of a number of transcriptomic sequences with cryptic homology, we also surveyed the gene complement of several homeobox classes in the *S. lamarcki* genome (Kenny et al. 2015) and a selection of other available lophotrochozoan genomes. We expand and modify Paps et al.’s (2015) system of lophotrochozoan homeobox classification, and compare and reconcile it with Morino et al.’s (2017) overlapping classification. We describe a surprising diversity of novel and difficult to classify homeobox genes in the transcriptomes of operculum regeneration, including members of a Spiralia-specific TALE-class gene radiation, a novel homeobox gene family restricted to lophotrochozoans, and an extremely divergent Hox gene.

## Materials and Methods

### Transcriptome

Animals were collected from East Sands in St. Andrews Bay, Fife, UK. Regeneration was induced as previously described (Szabó and Ferrier 2014). Total RNA was extracted from pooled mature opercular filaments (*n* = 22), noncalcifying 2 days-postamputation (dpa) (*n* = 19) and 6 dpa (*n* = 24) regenerating opercula using TRIsure, chloroform, and isopropanol (described in detail in Szabó, 2015). The samples were sequenced at the Wellcome Trust Centre for Human Genetics, Oxford, UK using the Illumina HiSeq2000 platform. Quality control was performed with FastQC v0.10.1, and adaptor removal, quality filtering and 3′ end trimming performed using the NGS-QC Toolkit v2.3 (Patel and Jain 2012) and assembled using Trinity (August 14, 2013 version) (Grabherr et al. 2011) with a default *k*-mer size of 25. Each sample pool produced >55 million paired-end reads, of which 80% were retained after quality control. The global assembly produced 360,107 contigs with a length >200 bases, with a mean length of 614 bases (SD = 865). This Transcriptome Shotgun Assembly project has been deposited at DDBJ/EMBL/GenBank under the accession GGGS00000000. The version described in this paper is the first version, GGGS01000000.

### Transcriptome and Genome Searches

Homeodomain sequences from *Branchiostoma floridae* and *Tribolium castaneum* were downloaded from HomeoDB2 (Zhong and Holland 2011a) and used along with homeodomain sequences from Kenny and Shimeld (2012) as queries for a tBLASTn (Altschul et al. 1997) search against the assembled transcriptomes. The resulting sequences were filtered for vertebrate and ciliate contamination using a BLASTp search against the NCBI database, and aligned against *B. floridae* and *T. castaneum* homeodomain sequences and previously described *S. lamarcki* homeodomain sequences (Hui 2008; McDougall et al. 2011; Kenny and Shimeld 2012). This alignment was used to produce a neighbor-joining phylogeny rooted using the yeast PHO2 homeodomain (see below).

Reads were quantified using BLASTn searches against the unassembled transcriptomes with a 95% identity cutoff and normalized using the mature transcriptome total read count.

For *S. lamarcki* homeobox families of interest, homologous sequences were collected from a relevant selection of annelid, brachiopod, mollusc, insect, deuterostome, cnidarian, and poriferan genomes using BLAST (see supplementary file 1, Sheet 7 for source details, Supplementary Material online) and from UniProt and the NCBI databases. For the noncanonical homeobox sequences, a query set of previously retrieved sequences (from Kenny and Shimeld, 2012, the regenerative transcriptomes, and Paps et al. 2015, including related canonical and noncanonical homeobox genes), were used to retrieve homeodomain sequences from the selected genomes by manual inspection of tBLASTn searches. Retrieved homeodomain sequences, having been putatively identified as not canonical on the basis of alignment, were added to the query pool and the process repeated until search saturation had been achieved. Full sequence details are included in supplementary file 1, Sheets 2–6, Supplementary Material online.

Where necessary, sequences were aligned using MAFFT v7.245 (Katoh and Standley 2013) and the alignment manually edited. The homeodomain (63 amino acids for TALE class homeodomains, 60 for others) or the homeodomain and five flanking sites either side for Hox/ParaHox sequences, was used to construct three sets of phylogenies (Neighbor-Joining, Maximum Likelihood and Bayesian).

### Alignment and Phylogenetic Analyses

The best-fit matrix of amino-acid evolution for each alignment was selected using ModelGenerator v0.85 (Keane et al. 2006) using four gamma categories. Where possible the recommended matrix and options were used in subsequent phylogenetic analyses; where the model was not supported, the default was used instead.

Neighbor-joining phylogenies were constructed in PHYLIP 3.695 (Felsenstein 1989) with 1000 bootstraps. A MEGA Analysis Options file was prepared in MEGA-Proto v7.0.26 for a maximum likelihood analysis using 1000 bootstraps, and run using MEGA-CC (Kumar et al. 2012). Bayesian analyses were run on the CIPRES Science Gateway (Miller et al. 2010), using MrBayes 3.2.6 (Ronquist and Huelsenbeck 2003) on XSEDE using a convergence diagnostic of 0.1.

A Python 2.7 script (supplementary file 6, Supplementary Material online) was written to map the support values (bootstraps from neighbor-joining and maximum likelihood analyses and posterior probabilities from Bayesian analyses) from nodes on each tree to equivalent nodes (where they exist) on a target tree. Trees were visualized in Figtree 1.4.2 (Rambaut 2007).

Clades were determined according to the following criteria; if any support value was above 70%, if they were reconstructed in all three analyses, or where informed by gene structure (e.g., TALE-IV), canonical orthology, or previous analyses (e.g., PRD-III). Homeobox families are referred to as “canonical” if they are listed in HomeoDB2 (Zhong and Holland 2011). Some clades were condensed based on less strict criteria to improve visibility (e.g., ambulacrarian Posterior Hox). Clade coloration is arbitrary and not meant to indicate a relationship (except in the case of the TALE-IV clades). Similarly, paralogue lettering, where present, is not intended to consistently imply direct orthology, though direct orthologues have been lettered accordingly where evident.

## Results

### The Homeodomain Content of Regenerative Transcriptomes

We analyzed transcriptomes of *Spirobranchus lamarcki* operculum regeneration for homeobox gene families (summarized in table 1). We identified 70 transcriptome component numbers (supplementary file 1, Sheet 1, Supplementary Material online), of which sixty could be assigned to “canonical” homeobox families (i.e., those listed on HomeoDB2—Zhong and Holland, 2011) by BLAST searches, protein sequence alignment, and homeodomain phylogenetic analyses (supplementary file 1, Supplementary Material online). Twenty-five of these were identical or near-identical to sequences previously described by Kenny and Shimeld (2012), and two were identical or near-identical to the *Dlx-a* and *Dlx-b* sequences previously described by McDougall et al. (2011). Three likely belong to the same multi-homeodomain gene (*Zfhx*). Three pairs were merged based on bridging genomic or developmental transcriptomic sequence. The remaining ten could not be placed in canonical clades, and a selection of detailed analyses were performed to classify these genes and to survey the various gene duplications in *S. lamarcki*.

**Table 1:** Summary of Homeobox-Containing Sequences Found in the *S. lamarcki* Regenerative Transcriptomes

Class	Family/Name	Class	Family/Name
ANTP:	***Antp***	POU:	Pou2*
	*BarH*		Pou3*
	*BarX*		*Pou4 A*
	Dbx*		*Pou4 B*
	*Dlx-a*^†^		*Pou6*
	*Dlx-b*^†^	PRD:	Gsc*
	*Emx A*		Hbn*
	*Emx B*		Otp A*
	*En*		*Otp B*
	*Msx*		Otx A*
	*Msxlx*		*Otx B*
	*Nk1a*		*Pax4/6 A*
	*Nk1b*		*Pax4/6 B*
	Nk2.1a*		***PRD-VIII***
	Nk2.1b*		*Prrx*
	*Nk2.2b*		*Shox*
	Nk5*		*Vsx B*
	Nk6*	SINE:	Six1/2*
	***Spiro-Nk***		*Six3/6 (B)*
	*Tlx E*		*Six4/5*
CERS:	Cers*	TALE:	*Irx A*
CUT:	Cmp*		***TALE-I A***
	Cux*		***TALE-I B***
	Onecut*		***TALE-X A***
HNF:	Hmbox*		***TALE-X B***
LIM:	Isl*		***TALE-XIII A***
	Lhx1/5*		***TALE-XIII B***
	Lhx2/9 A2*		Meis A*
	*Lhx2/9 B*		*Meis B*
	*Lmx*		Mkx A*
**(unclassified):**	***Lopx***		Pbx A*
ZF:	*Zfhx*		Pknox*
			Tgif A*

Note.—Sequences previously identified by McDougall et al. (2011) are marked with a dagger, and those previously identified by Kenny and Shimeld (2012) are marked with an asterisk. Difficult-to-classify genes are marked in bold, and those belonging to gene families or clades described herein are underlined.

### A Divergent *Antp* Hox Gene

**Fig. 1.**
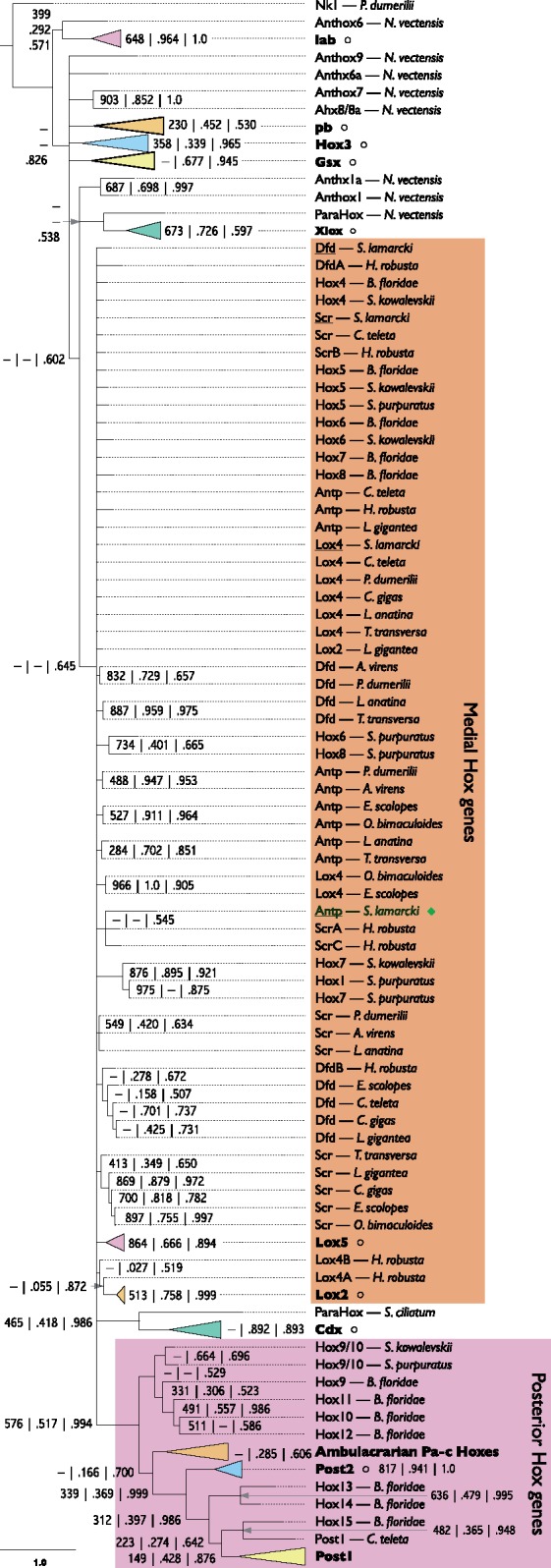
—Bayesian phylogeny of Hox and ParaHox homeodomains and flanking sequences from a selection of metazoan genomes, showing the basis for the identification of the divergent *Spirobranchus* Hox gene as *Antp*. Support values for each node are from neighbor-joining (out of 1000 bootstraps), maximum likelihood (proportion of 1000 bootstraps), and Bayesian (posterior probability) phylogenies (in order, separated by vertical bars or newlines). A dash indicates where a node is not present in the corresponding tree. Gene families that have been successfully reconstructed have been collapsed into colored triangles and a summary of their contents given in supplementary file 1, Supplementary Material online. *Spirobranchus* sequences (all underlined) are marked with a green diamond if found in the regenerative transcriptomes, and with a black circle if only found in the genome (collapsed families only). The scale bar indicates amino acid substitutions per site. Full sequence details are included in supplementary file 1, Sheet 2, Supplementary Material online. The original alignment is presented in supplementary file 7, Supplementary Material online. A full version of the Newick format tree is presented in supplementary file 2, Supplementary Material online. Annelid species: *S. lamarcki*, *Spirobranchus lamarcki*; *C. teleta*, *Capitella teleta*; *A. virens*, *Alitta virens*; *H. robusta*, *Helobdella robusta*; *P. dumerilii*, *Platynereis dumerilii*. Brachiopod species: *L. anatina*, *Lingula anatina*; *T. transversa*, *Terebratalia transversa*. Mollusc species: *C. gigas*, *Crassostrea gigas*; *L. gigantea*, *Lottia gigantea*; *E. scolopes*, *Euprymna scolopes*; *O. bimaculoides*, *Octopus bimaculoides*. Deuterostome species: *B. floridae*, *Branchiostoma floridae*; *S. kowalevski*, *Saccoglossus kowalevskii*; *S. purpuratus*, *Strongylocentrotus purpuratus*. Cnidarian species: *N. vectensis*, *Nematostella vectensis*. Poriferan species: *S. ciliatum, Sycon ciliatum*.

Among the difficult-to-classify genes was an unusual Hox/ParaHox-like gene. A broad selection of bilaterian Hox and ParaHox cluster protein sequences was collected and aligned (supplementary file 1, Sheet 2 and 7, Supplementary Material online), and a partially collapsed Bayesian phylogeny with support values added from equivalent neighbor-joining and maximum likelihood analyses was produced (fig. 1), based on the homeodomain and ten flanking positions (five from each side of the homeodomain). Candidate *S. lamarcki* orthologues were found in the whole genome sequence (Kenny et al. 2015) for all expected polychaete Hox (Fröbius et al. 2008) and ParaHox (Kulakova et al. 2008; Hui et al. 2009) families except *Antp* and *Post1*. Unfortunately, the analyses did not place *Dfd*, *Scr, Antp* and *Lox4* in distinct clades, but did place the unidentified gene in this undifferentiated *Hox4*/*5*/medial clade (fig. 1). On the basis of this placement and consistent support excluding it from other Hox/ParaHox clades, we conclude that the unidentified gene is most probably the missing *Antp* family gene.

**Fig. 2.**
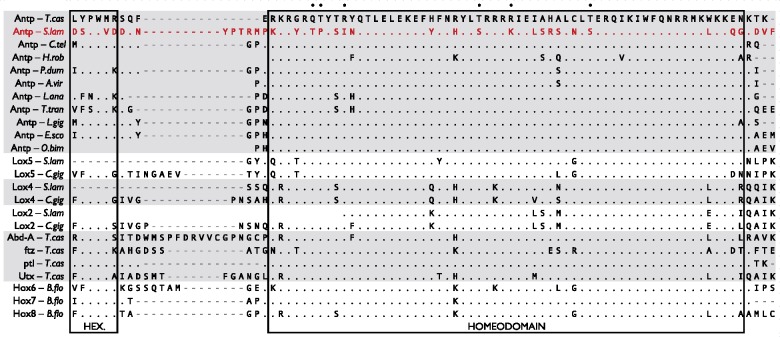
—Protein sequence alignment of hexapeptide, linker, homeodomain, and flanking region of medial Hox genes (Hox6–8 families) from a selection of bilaterians, demonstrating the degree of sequence divergence of *Spirobranchus* Antp (highlighted in red). Identities (full stop) are marked relative to the sequence of *Tribolium castaneum* Antp. Residue positions at which *Spirobranchus* Antp is the only variant sequence shown are marked with a black dot. Full sequence details are included in supplementary file 1, Sheet 2, Supplementary Material online. HEX., hexapeptide. Annelid sequences: *S.lam*, *Spirobranchus lamarcki*; *C.tel*, *Capitella teleta*; *H.rob*, *Helobdella robusta*; *P.dum*, *Platynereis dumerilii*; *A.vir*, *Alitta virens*. Brachiopod species: *L.ana*, *Lingula anatina*; *T.tra*, *Terebratalia transversa*. Mollusc species: *C.gig*, *Crassostrea gigas*; *L.gig*, *Lottia gigantea*; *E.sco*, *Euprymna scolopes*; *O.bim*, *Octopus bimaculoides*. Insect species: *T.cas*, *Tribolium castaneum*. Deuterostome species: *B.flo*, *Branchiostoma floridae*.

An alignment of this putative *S. lamarcki* Antp against other lophotrochozoan Antp proteins and a broader selection of other medial Hox sequences reveals that six residues in the homeodomain (marked by dots in fig. 2) are invariant across all included Hox sequences except the putative *S. lamarckiAntp*.

### TALE Class Homeodomains

**Fig. 3.**
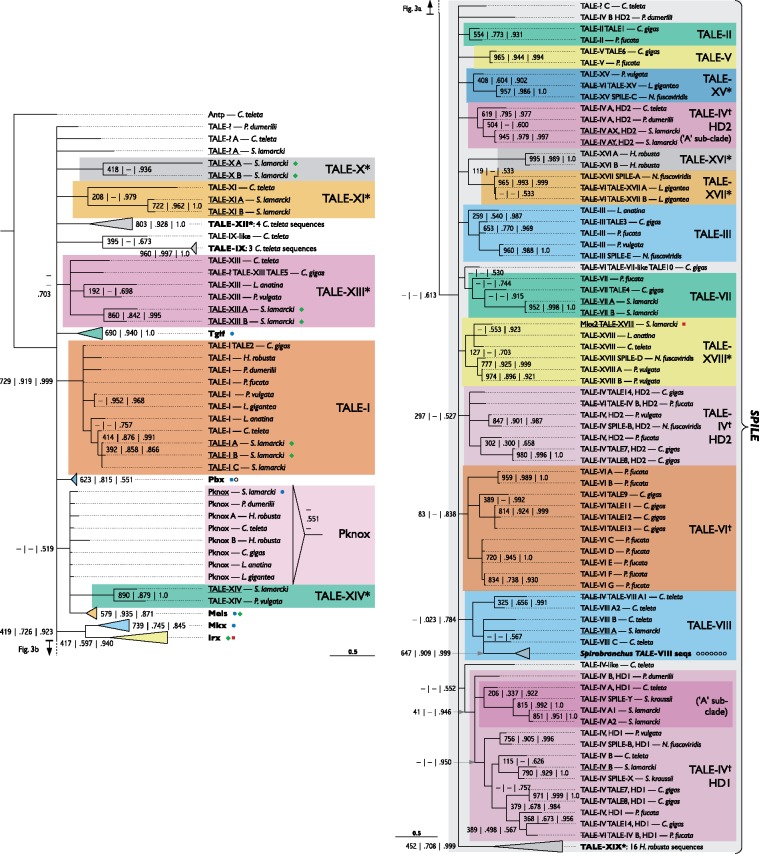
—Bayesian phylogeny of TALE class homeodomain sequences from a selection of lophotrochozoan genomes, showing the frequent duplication of canonical TALE class genes and the basis of our proposed revision to the TALE clade classification (Paps et al. 2015); split into two parts, a (left side) and b (right side). The SPILE clade (per Morino *et al.*, 2018) is marked by a grey box and labelled bracket in part b. Support values and formatting as in figure 1. In some cases, new families or family subsets containing several sequences all from a single genus have also been collapsed to aid visualization. Single genus families are highlighted in grey, but otherwise color selection is arbitrary, and not meant to indicate a relationship except in the case of the TALE-IV clades. Similarly, paralogue lettering, where present, is not intended to consistently imply direct orthology, though where evident, direct orthologues have been lettered accordingly. *S. lamarcki* sequences (all underlined) are marked with a green diamond if found in the regenerative transcriptomes, with a red square if found in the developmental transcriptome (Kenny and Shimeld 2012), and a blue dot if found in both. Collapsed families have their *S. lamarcki* gene complement indicated nearby with the same symbols as above, with an open circle indicating a gene that has been found only in the genome. New gene families suggested herein are marked with an asterisk. Gene families that have gained or lost sequences from Paps et al. (2015) are marked with a dagger. Where a gene has been reclassified from Paps et al. (2015) or Kenny and Shimeld (2012), the old classification is included but struck out. Established gene families that were successfully reconstructed in the neighbor-joining and/or maximum likelihood analyses but not the Bayesian analysis are marked by a “cartoon” clade (not to horizontal scale) and corresponding support values to the right-hand side. The scale bar indicates amino acid substitutions per site. Full sequence details are included in supplementary file 1, Sheet 5, Supplementary Material online. The original alignment is presented in supplementary file 8, Supplementary Material online. A full version of the Newick format tree is presented in supplementary file 3, Supplementary Material online. Annelid species: *S. lamarcki*, *Spirobranchus lamarcki*; *S. kraussi*, *Spirobranchus* (formerly *Pomatoleios*) *kraussi*; *C. teleta*, *Capitella teleta*; *H. robusta*, *Helobdella robusta*; *P. dumerilii*, *Platynereis dumerilii*. Brachiopod species: *L. anatina*, *Lingula anatina.* Mollusc species: *C. gigas*, *Crassostrea gigas*; *P. fucata*, *Pinctada fucata*; *L. gigantea*, *Lottia gigantea*; *N. fuscoviridis*, *Nipponacmea fuscoviridis*; *P. vulgata*, *Patella vulgata*. Insect species (only in collapsed clades): *Tribolium castaneum; Drosophila melanogaster*.

Thirteen transcriptomic homeodomain sequences had the three amino acid loop extension diagnostic of TALE (Three Amino-acid Loop Extension) class homeobox genes. Five of these were identical to previously described *S. lamarcki* canonical TALE-class genes: *Tgif, Pbx Pknox, Meis B*, and *Mkx1* (Kenny and Shimeld 2012). A further two of these could be classified on the basis of sequence phylogenies as other canonical TALE-class genes: *Meis A* and *Irx A* (fig. 3). Finally, six sequences were not obvious homologues of canonical TALE class families.

To classify these six sequences and to confirm the identifications of the other seven, we performed a deep recursive search for divergent TALE-class homeodomains in the available genomes of *S. lamarcki, Capitella teleta, Helobdella robusta*, *Platynereis dumerilii, Lingula anatina, Lottia gigantea*, and *Patella vulgata*. To these were added sequences from Paps et al.’s (2015) recent classification of spiralian TALE families, SPILE (Spiralian TALE) sequences from the NCBI database (Morino et al. 2017), and canonical TALE class family sequences.

An alignment of the homeodomains (supplementary file 8, Supplementary Material online) was used to construct a Bayesian phylogeny with support values added from equivalent neighbor-joining and maximum likelihood analyses (fig. 3). To accommodate all of these new and published sequences in a phylogenetically coherent framework, we propose an expansion and modification of Paps et al.’s (2015) system of nine lophotrochozoan TALE clades: TALE clades I–IX (See table 1 in Paps et al. 2015). We propose the reclassification of some members of two clades (TALE clades IV and VI), the addition of new orthologues to five clades (TALE clades I, III, IV, VII, and VIII), and the erection of ten new clades (TALE clades X–XIX), of which one may be the product of long-branch attraction (TALE-X), five are genus-specific (TALE clades X, XII, XIV, XVI, and XIX) and one contains a previously unclassified *Crassostrea* sequence (*TALE-XIII*). Our analysis suggests the sequence previously classified as an *Mkx* paralogue by Kenny and Shimeld (2012) belongs to TALE-XVIII. Seven sequences were found to be orphans or only weakly related to a clade. The unclassified transcriptome sequences were classed into TALE clades I, XIII, and X. A summary of the proposed changes and additions to the TALE classification is presented in table 2.

**Table 2:** Summary of Revisions to the TALE Classification System of Paps et al. (2015)

	Species	Origin	Sequence Name	HD1	HD2	Paps et al. (2015) Name	Original Classification
I	*S. lamarcki*	N	*TALE-I A♦, B♦, C*			—	—
	*C. teleta*	P	*TALE-I*			Ctel 1513294 24 8	Unchanged
	*H. robusta*	N	*TALE-I*			—	—
	*P. dumerilii*	N	*TALE-I*			—	—
	*L. anatina*	N	*TALE-I*			—	—
	*C. gigas*	P	*TALE-I TALE2*			Cgi TALE2	Unchanged
	*P. fucata*	P	*TALE-I*			Pfuc 24948 1 11659 JP	Unchanged
	*L. gigantea*	P	*TALE-I*			Lgig 1414665 30 1	Unchanged
	*P. vulgata*	N	*TALE-I*			—	—
II	*C. gigas*	P	*TALE-II TALE1*			Cgi TALE1	Unchanged
	*P. fucata*	P	*TALE-II*			Pfuc 13151 1 32296 JP/Pfuc 13478 1 32332 JP	Unchanged (HDs identical)
III	*L. anatina*	N	*TALE-III*			—	—
	*C. gigas*	P	*TALE-III TALE3*			Cgi TALE3	Unchanged
	*P. fucata*	P	*TALE-III*			Pfuc 98062 1 56909 JP	Unchanged
	*N. fuscoviridis*	M	*TALE-III SPILE-E*			—	—
	*P. vulgata*	N	*TALE-III*			—	—
IV	*S. lamarcki*	N	TALE-IV A1, A2, B	✓	✗	—	—
	*S. lamarcki*	N	*TALE-IV AX, AY*	F	✓	—	—
	*S. kraussi*	M	TALE-IV SPILE-X, SPILE-Y	✓	✗	—	—
	*C. teleta*	P	*TALE-IV A*	✓	✓	Ctel 1526117 32 9	Unchanged
	*C. teleta*	P	TALE-IV B	✓	✗	Ctel 1505080 24 4	Unchanged
	*P. dumerilii*	N	*TALE-IV B*	✓	W	—	—
	*P. dumerilii*	N	TALE-IV A	F	✓	—	—
	*C. gigas*	P	*TALE-IV TALE7, 8, 14*	✓	✓	Cgi TALE7, 8, 14	Unchanged
	*P. fucata*	P	TALE-IV A	✓	✓	Pfuc 1892 1 66137 JP	Unchanged
	*P. fucata*	P	*TALE-IV B*	✓	✓	Pfuc 6497 1 45448 JP	**TALE-VI**
	*N. fuscoviridis*	M	TALE-IV SPILE-B	✓	✓	—	—
	*P. vulgata*	N	*TALE-IV*	✓	✓	—	—
V	*C. gigas*	P	*TALE-V TALE6*			Cgi TALE6	Unchanged
	*P. fucata*	P	*TALE-V*			Pfuc 255 1 07443 JP	Unchanged
VI	*C. gigas*	P	*TALE-VI TALE9, 11-13*			Cgi TALE9, 11-13	Unchanged
	*P. fucata*	P	*TALE-VI A*			Pfuc 1442 1 22591 JP	Unchanged
	*P. fucata*	P	*TALE-VI B*			Pfuc 22569 1 62158 JP	Unchanged
	*P. fucata*	P	*TALE-VI C*			Pfuc 22555 1 40373 JP	Unchanged
	*P. fucata*	P	*TALE-VI D*			Pfuc 18402 1 40058 JP	Unchanged
	*P. fucata*	P	*TALE-VI E*			Pfuc 10095 1 38990 JP	Unchanged
	*P. fucata*	P	*TALE-VI F*			Pfuc 2547 1 30160 JP	Unchanged
	*P. fucata*	P	*TALE-VI G*			Pfuc 312 1 50785 JP	Unchanged
VII	*S. lamarcki*	N	*TALE-VII A, B*			—	—
	*C. gigas*	P	*TALE-VII TALE4*			Cgi TALE4	Unchanged
	*P. fucata*	P	*TALE-VII*			Pfuc 6013 1 23936 JP	Unchanged
VIII	*S. lamarcki*	N	*TALE-VIII A, B, C, D, E, F, G, H*			—	—
	*S. kraussi*	M	*TALE-VIII SPILE-Z*			—	—
	*C. teleta*	P	*TALE-VIII B (1-3?)*			Ctel 1505086 31 9/Ctel 1505698 31 9/Ctel 1499331 27 4	Unchanged (HDs identical)
	*C. teleta*	P	*TALE-VIII A1*			Ctel 1499505 38 4	**TALE-IV**
	*C. teleta*	M	*TALE-VIII A2, C*			—	—
IX	*C. teleta*	P	*TALE-IX A*			Ctel 1518266 30 6	Unchanged
	*C. teleta*	P	*TALE-IX B*			Ctel 1518128 28 9	Unchanged
	*C. teleta*	P	*TALE-IX C*			Ctel 1502937 32 5	Unchanged
X	*S. lamarcki*	N	*TALE-X A♦, B♦*			—	—
XI	*S. lamarcki*	N	*TALE-XI A, B*			—	—
	*C. teleta*	N	*TALE-XI*			—	—
XII	*C. teleta*	N/M	*TALE-XII A1, A2, A3, B*			—	—
XIII	*S. lamarcki*	N	*TALE-XIII A♦, B2♦*			—	—
	*C. teleta*	N	*TALE-XIII*			—	—
	*L. anatina*	N	*TALE-XIII*			—	—
	*C. gigas*	P	*TALE-XIII TALE5*			Cgi TALE5	TALE-?
	*P. vulgata*	N	*TALE-XIII*			—	—
XIV	*S. lamarcki*	N	*TALE-XIV*			—	—
	*P. vulgata*	N	*TALE-XIV*			—	—
XV	*L. gigantea*	P	*TALE-XV*			Lgig 1419427 48 9	**TALE-VI**
	*N. fuscoviridis*	M	*TALE-XV SPILE-C*			—	—
	*P. vulgata*	N	*TALE-XV*			—	—
XVI	*H. robusta*	N	*TALE-XVI A, B*			—	—
XVII	*L. gigantea*	P	*TALE-XVII A*			Lgig 1410135 44 3	**TALE-VI**
	*L. gigantea*	P	*TALE-XVII B*			Lgig 1410138 39 8	**TALE-VI**
	*N. fuscoviridis*	M	*TALE-XVII SPILE-A*			—	—
XVIII	*S. lamarcki*	N	*TALE-XVIII▪*			—	Mkx2
	*C. teleta*	M	*TALE-XVIII*			—	—
	*L. anatina*	N	*TALE-XVIII*			—	—
	*N. fuscoviridis*	M	*TALE-XVIII SPILE-D*			—	—
	*P. vulgata*	N	*TALE-XVIII A, B*			—	—
XIX	*H. robusta*	N	TALE-XIX A	✓	✓	—	—
	*H. robusta*	N	*TALE-XIX B-P (15 sequences)*			—	—
unclassified	*S. lamarcki*	N	*TALE-? A*			—	—
	*C. teleta*	M	*TALE-? A, C, TALE-IV-like, TALE-IX-like*			—	—
	*P. dumerilii*	N	*TALE-?*			—	—
	*C. gigas*	P	*TALE-VII-like TALE10*			Cgi TALE10	**TALE-VI**

Note.—In the Origin column, “N” denotes that the sequence is newly discovered by this analysis, “P” that the sequence was included in Paps et al.’s (2015) analysis, and “M” that the sequences were described by Morino et al. (2017). *S. lamarcki* sequences marked with green diamonds were found in the regenerative transcriptomes; those marked with red squares were described by Kenny and Shimeld (2012) in their developmental transcriptome. In genes with two homeodomains, a tick indicates the presence of a homeodomain. A cross indicates the absence, either through lack of sequence coverage or apparent homeodomain degradation. “F” indicates the presence of a truncated sequence due to lack of sequence coverage. “W” indicates a truncated homeodomain not due to lack of sequence coverage. An unusual *H. robusta* sequence with two homeodomains is highlighted in red. The Paps et al. (2015) name column refers to the identifying information given in Paps et al. (2015), and the Original classification column to the clade to which they were assigned by that analysis. Full sequence details are included in supplementary file 1, Sheet 5, Supplementary Material online. Annelid species: *S. lamarcki*, *Spirobranchus lamarcki*; *S. kraussi*, *Spirobranchus* (formerly *Pomatoleios*) *kraussi*; *C. teleta*, *Capitella teleta*; *H. robusta*, *Helobdella robusta*; *P. dumerilii*, *Platynereis dumerilii*. Brachiopod species: *L. anatina*, *Lingula anatina*. Mollusc species: *C. gigas*, *Crassostrea gigas*; *P. fucata*, *Pinctada fucata*; *L. gigantea*, *Lottia gigantea*; *N. fuscoviridis*, *Nipponacmea fuscoviridis*; *P. vulgata*, *Patella vulgata*.

**Fig. 4.**
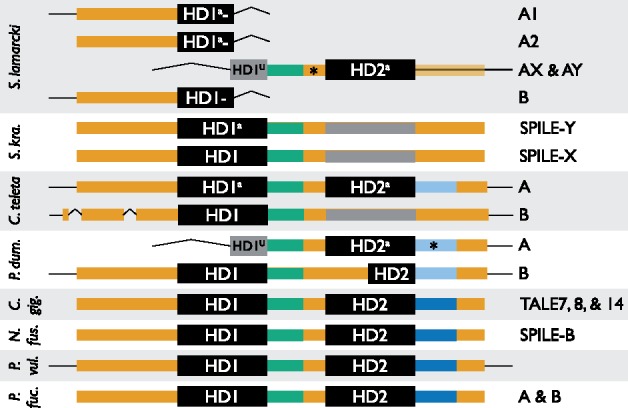
—A schematic of the sequence fragments of TALE clade IV (TALE-IV) family genes, showing the evidence for genes containing two TALE-class HDs. Noncoding sequence is indicated with a thin black line. Coding sequence is indicated with a thick colored line; semitransparent if the extent of the exonic sequence is not easily predictable. Green and blue regions represent areas of high sequence conservation C-terminal to each of the homeodomains. Light blue coloration represents regions where the sequence is recognisably homologous to the blue region but has substantially diverged. Regions that are unusually long relative to equivalent homologous regions are marked with an asterisk. Regions with apparent homology to homeodomains but which have degraded are represented with thick grey lines. Homeodomains are represented with boxes colored black if recognized by the NCBI Conserved Domain Search or grey otherwise. Half-size homeodomains are due to introns (*S. lamarcki* AX and AY, *P. dumerilii* A) or truncated homeoboxes (*P. dumerilii* B). Homeodomains are marked “a” if they belong to the A/annelid-only subclade (see fig. 3) or “U” if they were too short to be identified using the phylogeny. Where two or more paralogues have structures equivalent for the purposes of this diagram, they have been amalgamated and listed to the right. Not to scale. Annelid species: *S. lamarcki*, *Spirobranchus lamarcki*; *S. kraussi*, *Spirobranchus kraussi* (formerly *Pomatoleios*); *C. teleta*, *Capitella teleta*; *P.dum.*, *Platynereis dumerilii*. Mollusc species: *C. gig.*, *Crassostrea gigas*; *P. fuc.*, *Pinctada fucata*; *N. fus.*, *Nipponacmea fuscoviridis*; *P. vul.*, *Patella vulgata*.

In the course of manually inspecting sequences for alignment, we observed that most TALE-IV sequences have two TALE-class homeodomains. The available evidence for TALE-IV gene structure is summarized in figure 4. TALE-IV sequences with a single homeodomain could be the result of incomplete sequence coverage, though all contain regions that appear to be degraded homeodomains. Regions with homology to the PADRE domain described by Paps et al. 2015 in TALE clades VI and VII are in all members of TALE clades XV, XVII and XVIII with adequate sequence coverage (supplementary file 1, Sheet 10 and 11, Supplementary Material online). The new members of TALE-VII (*S. lamarckiTALE-VII A* and *B*) do not have enough coverage to confirm the presence of a PADRE domain.

### PRD Class Homeodomains

**Fig. 5.**
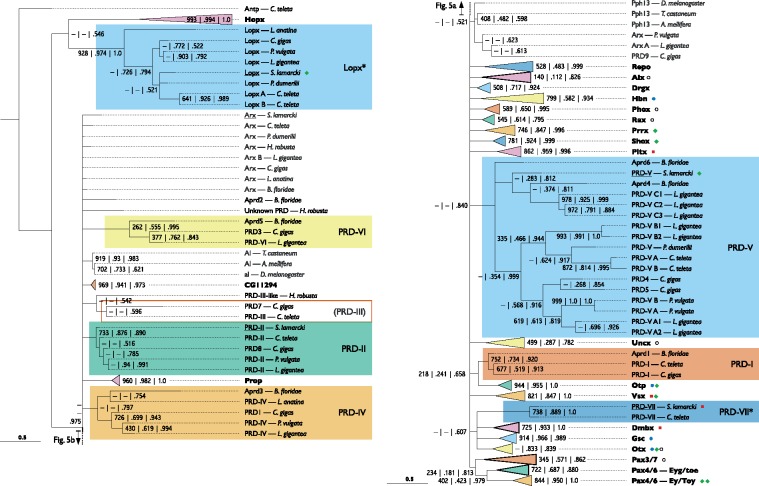
—Bayesian phylogeny of PRD class homeodomain sequences from a selection of bilaterian genomes, and the new unclassified *Lopx* gene family. Formatting as in figures 1 and 3. A previously reconstructed clade (PRD-III from Paps et al*.* 2015), which is topologically intact but does not meet the clade definition criteria is indicated with an empty box. Full sequence details are included in supplementary file 1, Sheet 4, Supplementary Material online. The original alignment is presented in supplementary file 9, Supplementary Material online. A full version of the Newick format tree is presented in supplementary file 4, Supplementary Material online. Annelid species: *S. lamarcki*, *Spirobranchus lamarcki*; *C. teleta*, *Capitella teleta*; *H. robusta*, *Helobdella robusta*; *P. dumerilii*, *Platynereis dumerilii*. Brachiopod species: *L. anatina*, *Lingula anatina*. Mollusc species: *C. gigas*, *Crassostrea gigas*; *L. gigantea*, *Lottia gigantea*; *P. vulgata*, *Patella vulgata*. Insect species: *A. mellifera*, *Apis mellifera*; *D. melanogaster*, *Drosophila melanogaster*; *T. castaneum*, *Tribolium castaneum*. Deuterostome species: *B. floridae*, *Branchiostoma floridae*.

We identified ten transcriptomic sequences as canonical PRD-class genes: *Prrx, Shox, Otp B, Otx B, Vsx B, Pax4*/*6 A* and *B*, and four identical or near-identical to previously described *S. lamarcki* sequences: *Gsc, Hbn, Otp A*, and *Otx A* (Kenny and Shimeld 2012). Two sequences were also identified which could not be placed in canonical PRD-class gene families. One of these was matched by BLAST to sequences that had been automatically identified as *ceh-37*, one of the *Caenorhabditis elegans* paralogues of *Otx*, but appeared to share little similarity with the original *ceh-37* gene. The other was matched by BLAST searches to *B. floridae Aprd6.* To classify these genes, we aligned putative and previously identified PRD-class homeodomains from a selection of annelid, brachiopod, mollusc, insect, and cephalochordate genomes (supplementary file 1, Sheet 4 and 9, Supplementary Material online). This homeodomain alignment was used to produce a Bayesian phylogeny with support values added from equivalent neighbor-joining and maximum likelihood analyses (fig. 5).

This phylogeny successfully reconstructed all canonical PRD-class clades (except *Arx*) and the same noncanonical PRD Clades as Paps et al. (2015) (PRD Clades I–VI), although PRD-III did not meet the clade definition criteria. In addition, a further clade (PRD VII) was resolved, including a previously described *S. lamarcki* sequence, *Prd-like* (Kenny and Shimeld 2012).

### A Novel Unclassified Homeobox Gene Family

**Fig. 6.**
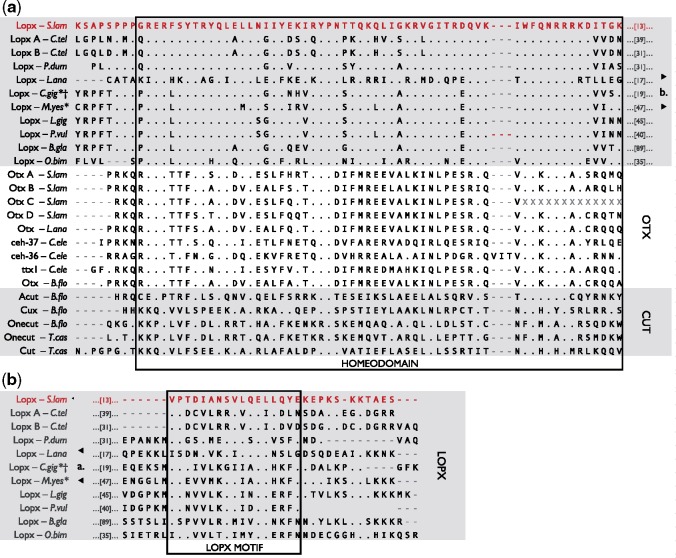
—Sequence alignment of Lopx homeodomain and N-terminal flanking region (*a*) and a C-terminal conserved motif unique to Lopx proteins (*b*) from a selection of lophotrochozoan species, compared with gene families/classes that *Lopx* genes have been mistaken for by automatic annotation pipelines (Otx/ceh-37—marked with asterisks) and in general homeodomain trees (CUT class—marked with dagger). Identities (full stops) are marked relative to the sequence of *Spirobranchus lamarcki* Lopx. The *S. lamarcki* Lopx sequence is highlighted in red. Full sequence details are included in supplementary file 1, Sheet 6, Supplementary Material online. Annelid species: *S.lam*, *Spirobranchus lamarcki*; *C.tel*, *Capitella teleta*; *P.dum*, *Platynereis dumerilii*. Brachiopod species: *L.ana*, *Lingula anatina*. Mollusc species: *C.gig*, *Crassostrea gigas*; *L.gig*, *Lottia gigantea*; *P.vul*, *Patella vulgata*; *M.yes*, *Mizuhopecten yessoensis* (syn. *Patinopecten yessoensis*); *B.gla*, *Biomphalaria glabrata*; *O.bim*, *Octopus bimaculoides*. Ecdysozoan species: *C.ele*, *Caenorhabditis elegans*; *T.cas*, *Tribolium castaneum*. Deuterostome species: *B.flo*, *Branchiostoma floridae*.

The putative *ceh-37* genes grouped into their own strongly supported clade separate from all PRD-class gene families except the highly divergent *Hopx.* We therefore propose a new gene family, named *Lopx* (LOPhotrochozoan only homeobox). An alignment of the homeodomain and some flanking sequence of these proteins against sequences which they have previously been putatively identified with, as well as a conserved motif unique to *Lopx* genes, illustrates the distinctive nature of the *Lopx* family (fig. 6).

### *Nk*, *Msx*, *Lbx*, and *Tlx* Families

**Fig. 7.**
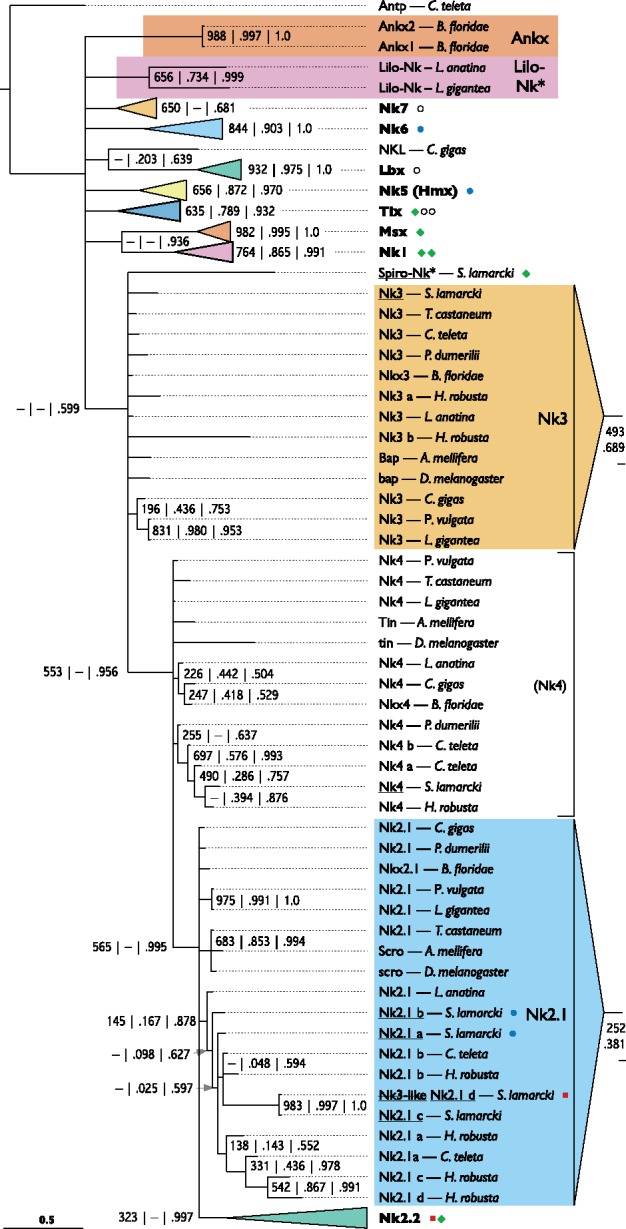
—Bayesian phylogeny of Nk, Msx, Tlx, and Lbx homeodomain sequences from a selection of bilaterian genomes, showing the various *Spirobranchus* gene duplications and the Spiro-Nk orphan. Formatting as in figures 1 and 3. Full sequence details are included in supplementary file 1, Sheet 3, Supplementary Material online. The original alignment is presented in supplementary file 10, Supplementary Material online. A full version of the Newick format tree is presented in supplementary file 5, Supplementary Material online. Annelid species: *S. lamarcki*, *Spirobranchus lamarcki*; *C. teleta*, *Capitella teleta*; *H. robusta*, *Helobdella robusta*; *P. dumerilii*, *Platynereis dumerilii*. Brachiopod species: *L. anatina*, *Lingula anatina*. Mollusc species: *C. gigas* = *Crassostrea gigas*; *L. gigantea*, *Lottia gigantea*; *P. vulgata*, *Patella vulgata*. Insect species: *A. mellifera*, *Apis mellifera*; *D. melanogaster*, *Drosophila melanogaster*; *T. castaneum*, *Tribolium castaneum*. Deuterostome species: *B. floridae*, *Branchiostoma floridae*.

We identified seven sequences from the transcriptomes as members of canonical Nk families: *Nk1a, Nk1b*, *Nk2.2b*, and four identical or nearly identical to previously described *S. lamarcki* sequences: *Nk2.1a*, *Nk2.1b*, *Nk5*, and *Nk6* (Kenny and Shimeld 2012). We also identified an eighth sequence similar to *Nk* genes that could not be placed in a canonical family. To classify the known sequences and profile Nk family gene duplication in *S. lamarcki*, we aligned putative and previously identified *Nk1-7, Msx, Lbx*, and *Tlx* homeodomain sequences from the genomes of a selection of annelid, brachiopod, mollusc, insect, and cephalochordate species, (supplementary file 1, Sheet 3 and 10, Supplementary Material online) including the noncanonical *C. gigas NKL* gene and the amphioxus *Ankx* genes. This alignment was used to produce a Bayesian phylogeny with support values added from equivalent neighbor-joining and maximum likelihood analyses (fig. 7). All clades except *Nk2.1*, *Nk3*, and *Nk4* were successfully reconstructed. Our analysis does not suggest a common origin of all divergent lophotrochozoan *Nk* genes except those from *L. anatina* and *L. gigantea*, leading us to name them *Lilo-Nk* (i.e., *Lingula*-*Lottia* Nk). Although the unidentified *Spirobranchus* Nk gene is located close to the Nk3 family members in figure 7, it has a clearly different sequence, leading us to name it *Spiro-Nk*. The phylogeny also indicates that *S. lamarcki Nk3-like* (Kenny and Shimeld 2012) should be reclassified as an *Nk2.1* paralogue (*Nk2.1d*).

## Discussion

Given the generally conservative nature of annelid genome evolution relative to many other animal lineages (Raible et al. 2005; Hui et al. 2009, 2012; Ferrier 2012), the regenerative transcriptome of *S. lamarcki* contains a surprising diversity of noncanonical and difficult-to-classify homeobox genes from several classes, including six non-SPILE TALE class genes, a PRD class gene, an Nk gene, a divergent Hox gene, and one other unclassified gene. To classify these genes, we undertook an in-depth survey of the related homeobox gene complement of the genome of *S. lamarcki* (Kenny et al. 2015) and a selection of other available lophotrochozoan genomes.

*Spirobranchus* *lamarcki* shows signs of unusual Hox gene evolution and deployment. We identified normal orthologues of nine of the 11 expected Hox families, missing *Antp* and *Post1*. Based on our phylogenetic analysis, we conclude that a difficult-to-classify Hox gene found in our transcriptome data is likely to be a highly divergent *Antp* orthologue, and that *S. lamarcki* has potentially lost *Post1*. This divergent *Antp* is the only Hox gene yet found to be expressed in *S. lamarcki* in any context, including in a previous developmental transcriptome (Kenny and Shimeld 2012). This paucity of Hox expression is surprising given the known expression of a wide variety of Hox genes in the development of *Chaetopterus* (Irvine and Martindale 2000), two nereids (Kulakova et al. 2007), and *Helobdella* (Kourakis and Martindale 2001; Gharbaran et al. 2012; Gharbaran et al. 2014), and the caudal regeneration of nereids (Pfeifer et al. 2012; Novikova et al. 2013) and *Capitella* (de Jong and Seaver 2016), as well as in regeneration more generally (Wang et al. 2009; Novikova et al. 2016). One intriguing possibility is that this unusual lack of Hox deployment could somehow be related to *S. lamarcki*’s poor capacity for main body axis regeneration compared with many other annelids (Bely et al. 2014) and possibly, to its blastema-less operculum regeneration (Szabó and Ferrier 2014). The expression of Hox genes in *S. lamarcki* embryogenesis, larval development and a range of regenerative processes is thus an important avenue for future research to attempt to resolve this currently puzzling anomaly.

We undertook an extensive survey of the canonical and noncanonical TALE and PRD class homeodomains in the *S. lamarcki* genome, which we integrated into Paps et al.’s (2015) TALE and PRD clade nomenclature system. Our results offer a substantial expansion on previous classifications of these noncanonical genes, with the inclusion and classification of many more sequences and surveying previously unsampled clades, including brachiopods. To fulfil the purpose of identifying the difficult-to-classify genes in the *S. lamarcki* regenerative transcriptomes, we elected to sample only then-available lophotrochozoan (sensu stricto) genomes, excluding the TALE and PRD sequences from Platyhelminthes and Rotifera included in earlier analyses (Paps et al*.* 2015; Morino et al. 2017). Although this is a limitation, the comparative paucity of platyhelminth and rotifer sequences retrieved by these analyses (and the absence of TALE or PRD clades with no trochozoan gene members) suggests that the most radical homeobox expansions might be restricted to the molluscs and annelids. Our Bayesian analysis reconstructs, though with low support, the monophyletic SPILE (SPIralian taLE) gene clade erected by Morino et al*.* (2017), though our finding of six non-SPILE TALE sequences in the regenerative transcriptome highlights the potential importance of non-SPILE as well as SPILE TALEs in spiralian development.

A serious issue with the survey of noncanonical TALE genes in Spiralia is the unreliability of searches in producing an exhaustive data set; for example, three separate searches of the genome of *C. teleta* ([Paps et al. 2015; Morino et al. 2017], present study) each produced a different set of genes, with each survey identifying homeodomains the others had missed, but missing some themselves. This may be an artefact of the query set used by each study, indicating the paramount importance of a diverse, constantly updated, and recursive query pool, and of repeating searches of previously surveyed genomes to make use of expansions to the query pool. In addition, there is a need for ever greater taxon sampling, including undersampled annelid (e.g., Amphinomidae [Mehr et al. 2015]) and mollusc (e.g., Cephalopoda [Albertin et al. 2015]) clades, and the recently published nemertean and phoronid draft genomes (Luo et al. 2018).

The clades we propose each inspire rather different degrees of confidence. Some, like TALE clades I–III, have been reconstructed in phylogenies produced from various alignments, and in multiple phylogenetic analyses of the same alignment, whereas others (e.g., TALE-X) appear on sequence inspection to be products of long branch attraction, only just meet our naming criteria (e.g., TALE-XIII), or were inconsistently reconstructed between analyses (e.g., TALE clades VI and XVIII). We suggest that the fragility or robustness of a clade between alignments and methodologies might be a better indication of confidence than the phylogenetic support values.

Another issue with some TALE homeodomain phylogenies is the problem of consistently determining what qualifies as a clade; although Morino et al.’s (2017) analysis diverges from ours in only a single place where equivalent data are included (their *CtTALEHD40* was placed in TALE-XVIII with *NfSPILE-D* by our analysis), the same nodes could not be confidently dubbed clades, having inconsistent depths and support values. We found Bayesian phylogeny to be indispensable in informing the naming of clades because of its propensity to collapse uninformative nesting of nodes into large parallel nodes, each containing usually only well-supported clades.

The chosen criteria for clade definition are not particularly stringent but were selected because they allow for the replication of previously described noncanonical clades (TALE clades I–IX and PRD Clades I–VI) and canonical gene families, and place both new and old noncanonical clades on a basis of confidence comparable to that of canonical families within the context of homeodomain phylogeny. However, the determination of orthology in canonical families is often based on additional data from outside the homeodomain, and consequently the TALE and PRD clades should not be seen as robust orthology groups until further evidence is collected (as with *Lopx* and TALE clades IV, VI, VII, XV, XVII, and XVIII). Some (e.g., TALE-X, and the inclusion of cephalochordate sequences in PRD Clades I, IV, V, and VI) should be treated with particular caution as potential products of long-branch attraction, and the entire system of nomenclature will possibly be subject to further revision as more data become available.

Despite the difficulties with topological variability and varying confidence levels, our analysis supports the value of trying to detect orthology within the noncanonical TALEs. Characteristics of the genes outside of the homeodomain sequence (e.g., presence/absence of multiple homeodomains) supports the idea that there are taxonomically deep and discernible orthologies beyond the monophyletic SPILE/non-SPILE distinction made by Morino et al. (2017). One disadvantage of treating the SPILE genes as a homogenous clade is that this approach could miss potentially interesting information about the (possibly extreme) degree of evolutionary flexibility exhibited by these genes. For example, our analysis indicates that *NfSPILE-B*, *SkSPILE-X*, and *SkSPILE-Y* (Morino et al. 2017) are all members of TALE-IV, but have diverged in potentially interesting ways. Although *NfSPILE-B* is a typical two-homeodomain TALE-IV protein (fig. 4), *SkSPILEsX* and *Y* each have only one intact homeodomain, but both appear to possess a degraded homeodomain C-terminal to the intact one.

The potential orthology between *NfSPILE-B* and *SPILE-X/Y* and paralogy between *SkSPILEs X* and *Y* sheds an interesting new light on the similarities and dissimilarities between their early expression domains. Interpretation of Morino et al.’s (2017) results could also be shaped by the placement of *NfSPILEs A* and *C* in well-supported gastropod-only clades (TALE clades XVII and XV, respectively), indicating that these genes might be comparatively “new” (either in origin or by strong sequence divergence) compared with *NfSPILE-D* and *E*, both of which belong to Spiralia-wide clades.

The identification of genes containing two homeoboxes (some members of the TALE-IV clade—figs. 3b and 5) is another unusual characteristic of the noncanonical spiralian TALE genes, highlighting the value of careful manual curation alongside automated homeodomain searches. Curiously, a *H. robusta* sequence (*TALE-XIX A*) also seems to have acquired a second homeobox independently of the presumed TALE-IV pro-orthologue. A multi-homeobox state has not previously been observed for any TALE class genes, and is only rarely seen in some other animal homeobox gene classes, such as *Hdx* (POU class), *dve/Compass* (CUT class), *Zfhx* and *Zhx/Homez* (ZF class), *Muxa* and *Muxb* (orphan genes in amphioxus), and *Dux* genes in mammals (PRD class) (Booth and Holland 2007; Takatori et al. 2008; Zhong and Holland 2011b).

The difficulties discussed above of finding divergent TALE sequences using previously known homeodomain sequences and of detecting orthology groups, the inconsistent presence/absence of direct orthologues between relatively close relatives (e.g., *P. vulgata* and *L. gigantea*, both true limpets), and the prevalence of single-species-only clades of divergent TALE genes in particular species (e.g., *Capitella* and *Helobdella*) or other taxonomically restricted orthology groups, indicate that these genes undergo rapid and relatively unconstrained duplication, sequence divergence, and loss. In this sense, the noncanonical TALE clade homeobox gene expansion appears to be unusual in the evolutionary use of homeobox genes. Other radical expansions of homeobox complements have previously been reported, for example of Lepidoptera *Hox3* (Chai et al. 2008) and human *Dux* genes (Booth and Holland 2007), reviewed in Holland et al. (2017), but these are smaller in taxonomic scope and sequence diversity. The spiralian TALE expansion is the largest and most diverse taxonomically restricted homeobox expansion yet described.

In addition to its substantial TALE expansion, *S. lamarcki* has three noncanonical PRD-class genes, only one of which is a member of one of the PRD clades described by Paps et al. (2015) (*PRD-II*). Another, previously named *PRD-like* (Kenny and Shimeld 2012), is expressed during development and is only otherwise found in *Capitella* (*PRD-VII*). The third, found in the regeneration transcriptomes, belongs to a new but weakly supported clade (PRD-VIII). Our phylogeny suggests that some of Paps et al.’s PRD clades (namely I, IV, V, and VI) include cephalochordate *Aprd* genes, raising the possibility that the bilaterian ancestor had four PRD pro-orthologues, which, being lost in most deuterostomes and the Ecdysozoa, were previously unidentified as homeobox families.

**Fig. 8.**
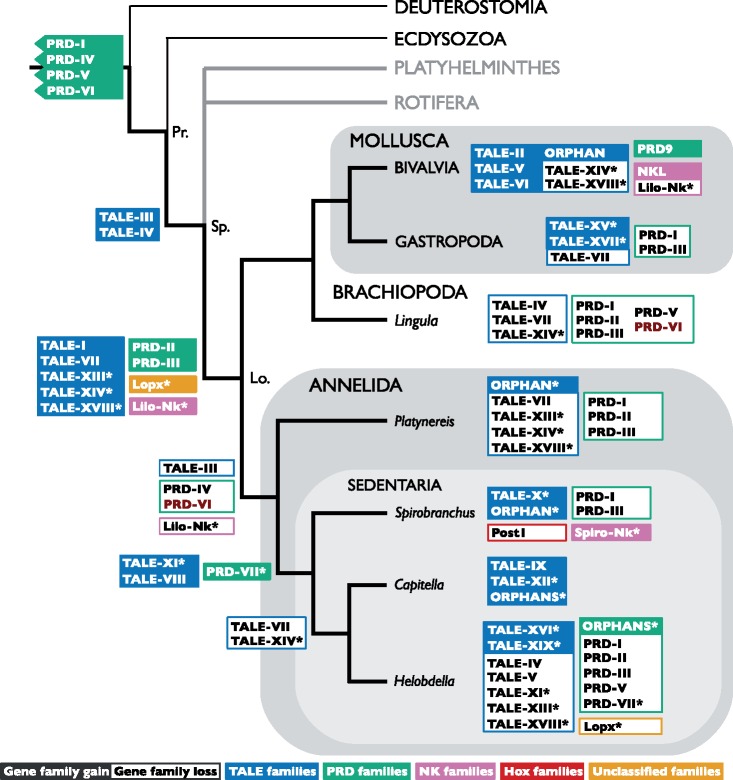
—Cladogram of the Bilateria, focusing on the annelids, summarizing the minimum gene family gain and loss events necessary to explain the pattern of gene presence and absence in the species surveyed, for TALE class genes (blue), PRD class genes (green), *Nkx* genes (pink), Hox genes (red), and unclassified genes (orange). White text on a colored background indicates a putative gene gain event; black text on a white background with a colored border indicates a putative gene loss event. The only gain or loss event influenced by the internal topology of the Lophotrochozoa is marked in dark red (PRD-VI). New gene families suggested herein are marked with an asterisk. Clades not sampled in these analyses are marked with grey lines. Clades from which sequences were included but not extensively surveyed in our work and with severely limited taxonomic sampling are marked by a thin black line. The Protostomia, Spiralia, and Lophotrochozoa clade nodes are marked Pr., Sp., and Lo., respectively. The topology of the cladogram is adapted from data in Weigert et al. (2014) and Luo et al. (2018), and the position of some gain/loss events from Paps et al. (2015). Clades not sampled here or in Paps et al. (including Phoronida, Nemertea, Entoprocta, and Gastrotricha) have been omitted to aid comparison with Paps et al. figure 4. For collapsed clades with more than one sampled species (i.e., Bivalvia and Gastropoda), gene gains are marked if they have been found in any of the species in that group, but gene losses marked only if they have not been identified in any. Changes to canonical families are only marked for *S. lamarcki*.

A cladogram depicting the most parsimonious pattern of gene gain and loss necessary to explain the distribution of genes found in this analysis is presented in figure 8. It is noticeable that the largest gene gain cluster appears to be at the trochozoan node, particularly in the TALE clades, and that no gene gain event is synapomorphic to any of the major sampled phyla. However, any attempt to discern a pattern from this information must consider a number of caveats, including the inconsistent clade collapsing, and sampling depth and breadth, and this pattern will no doubt change as taxon sampling (particularly those entirely omitted from the cladogram) improves. Assuming no major disruption to the TALE and PRD nomenclatures, gene gains will tend to move earlier and gene losses more recent. Some species (particularly *Platynereis, Helobdella* and *Lingula*) seem to have undergone slightly higher levels of loss relative to the other species sampled here.

Homeobox genes are instrumental in the orchestration of a huge variety of developmental mechanisms, including in regeneration and biomineralization. The operculum regeneration transcriptomes contain a broad selection of canonical ANTP-, CUT-, LIM-, POU-, PRD-, SINE-, and TALE-class genes, many of them accompanied by paralogues. Additionally, we report the expression of a surprising number of novel homeobox genes, including a previously unidentified homeobox gene family (*Lopx*), members of rapid taxonomically restricted homeobox expansions with cryptic orthology (*TALE IA* and *B*, *XA* and *B*, *XIIIA* and *B*, and *PRD-V*) and highly divergent canonical homeobox genes (*Antp* and *Spiro-Nk*). This diversity of divergent homeobox genes, considered in combination with the absence of some expected gene families (i.e., other Hox genes), indicates that *S. lamarcki* is unusual compared with previous surveys of regeneration. Further unbiased surveys of expression in new regenerative models are necessary to determine whether the *S. lamarcki* operculum is an isolated example of divergence or represents a previously hidden but widespread diversity of homeobox deployment in regeneration.

The historical study of the deep homology of homeobox gene families, and the relations between ancient sequence, synteny, regulatory, and functional conservation, have been of cardinal importance to the understanding of animal ontology and evolution produced by the field of Evo-Devo. However, the Spiralia seem to possess an unprecedented diversity of relatively unconstrained and taxonomically restricted homeobox genes in addition to the expected complement of bilaterian homeobox families. Understanding what these genes do, why they are gained and lost so readily, and why they diverge so quickly in the meantime, could help elucidate why the Spiralia are so phyletically and morphologically diverse (Giribet 2008).

## Supplementary Material

Supplementary Data

## References

[evy144-B1] Agata K, Saito Y, Nakajima E. 2007 Unifying principles of regeneration I: epimorphosis versus morphallaxis. Dev Growth Differ. 49(2):73–78. 17335428 10.1111/j.1440-169X.2007.00919.x

[evy144-B2] Albertin CB, et al 2015 The octopus genome and the evolution of cephalopod neural and morphological novelties. Nature 524(7564):220. 26268193 10.1038/nature14668 PMC4795812

[evy144-B3] Altschul SF, et al 1997 Gapped BLAST and PSI-BLAST: a new generation of protein database search programs. Nucleic Acids Res. 25(17):3389–3402. 9254694 10.1093/nar/25.17.3389 PMC146917

[evy144-B4] Alvarado AS, Tsonis PA. 2006 Bridging the regeneration gap: genetic insights from diverse animal models. Nat Rev Genet. 7(11):873–884. 17047686 10.1038/nrg1923

[evy144-B5] Balavoine G. 2014 Segment formation in annelids: patterns, processes and evolution. Int J Dev Biol. 58(6-8):469–483. 25690963 10.1387/ijdb.140148gb

[evy144-B6] Bely AE. 2006 Distribution of segment regeneration ability in the Annelida. Integr Comp Biol. 46(4):508–518. 21672762 10.1093/icb/icj051

[evy144-B7] Bely AE. 2014 Early events in annelid regeneration: a cellular perspective. Integr Comp Biol. 54(4):688–699. 25122930 10.1093/icb/icu109

[evy144-B8] Bely AE, Zattara EE, Sikes JM. 2014 Regeneration in spiralians: evolutionary patterns and developmental processes. Int J Dev Biol. 58(6-8):623–634. 25690976 10.1387/ijdb.140142ab

[evy144-B9] Ben Khadra Y, Said K, Thorndyke M, Martinez P. 2014 Homeobox genes expressed during echinoderm arm regeneration. Biochem Genet. 52(3-4):166–180. 24309817 10.1007/s10528-013-9637-2

[evy144-B10] Boilly B, Boilly‐Marer Y, Bely AE. 2017 Regulation of dorso‐ventral polarity by the nerve cord during annelid regeneration: a review of experimental evidence. Regeneration 4(2):54–68. 28616245 10.1002/reg2.78 PMC5469730

[evy144-B11] Bok MJ, Porter ML, ten Hove HA, Smith R, Nilsson D-E. 2017 Radiolar eyes of serpulid worms (Annelida, Serpulidae): structures, function, and phototransduction. Biol Bull. 233(1):39–57. 29182501 10.1086/694735

[evy144-B12] Booth HAF, Holland PWH. 2007 Annotation, nomenclature and evolution of four novel homeobox genes expressed in the human germ line. Gene 387(1-2):7–14. 17005330 10.1016/j.gene.2006.07.034

[evy144-B13] Boyle MJ, Yamaguchi E, Seaver EC. 2014 Molecular conservation of metazoan gut formation: evidence from expression of endomesoderm genes in *Capitella teleta* (Annelida). EvoDevo 5:39. 25908956 10.1186/2041-9139-5-39 PMC4407770

[evy144-B14] Bubel A, Thorp CH. 1985 Tissue abscission and wound healing in the operculum of *Pomatoceros lamarckii* Quatrefages (Polychaeta: Serpulidae). J Zool. 1(1):95–143.

[evy144-B16] Chai C-L, et al 2008 A genomewide survey of homeobox genes and identification of novel structure of the Hox cluster in the silkworm, *Bombyx mori*. Insect Biochem Mol. 38(12):1111–1120. 10.1016/j.ibmb.2008.06.008 19280701

[evy144-B17] Christodoulou F, et al 2010 Ancient animal microRNAs and the evolution of tissue identity. Nature 463(7284):1084–1088. 20118916 10.1038/nature08744 PMC2981144

[evy144-B18] de Jong DM, Seaver EC. 2016 A stable thoracic Hox code and epimorphosis characterize posterior regeneration in *Capitella teleta*. Plos ONE 11(2):e0149724. 26894631 10.1371/journal.pone.0149724 PMC4764619

[evy144-B19] Dray N, et al 2010 Hedgehog signaling regulates segment formation in the annelid *Platynereis*. Science 329(5989):339–342. 20647470 10.1126/science.1188913 PMC3182550

[evy144-B20] Felsenstein J. 1989 PHYLIP - Phylogeny Inference Package (Version 3.2). Cladistics 5:164–166.

[evy144-B21] Ferrier DEK. 2012 Evolutionary crossroads in developmental biology: annelids. Development 139(15):2643–2653. 22782719 10.1242/dev.074724

[evy144-B22] Fröbius AC, Matus DQ, Seaver EC. 2008 Genomic organization and expression demonstrate spatial and temporal Hox gene colinearity in the lophotrochozoan *Capitella* sp. I. PLoS One 3(12):e4004. 19104667 10.1371/journal.pone.0004004 PMC2603591

[evy144-B200] Gardiner DM, Bryant SV. 1996 Molecular mechanisms in the control of limb regeneration: the role of homeobox genes. Int J Dev Biol. 40(4):797–805. 8877453

[evy144-B23] Gersch RP, Lombardo F, McGovern SC, Hadjiargyrou M. 2005 Reactivation of Hox gene expression during bone regeneration. J Orthop Res. 23(4):882–890. 16023004 10.1016/j.orthres.2005.02.005

[evy144-B24] Gharbaran R, Aisemberg GO, Alvarado S. 2012 Segmental and regional differences in neuronal expression of the leech Hox genes *Lox1* and *Lox2* during embryogenesis. Cell Mol Neurobiol. 32(8):1243–1253. 22569741 10.1007/s10571-012-9849-8 PMC11498516

[evy144-B25] Gharbaran R, Alvarado S, Aisemberg GO. 2014 Regional and segmental differences in the embryonic expression of a putative leech Hox gene, Lox2, by central neurons immunoreactive to FMRFamide-like neuropeptides. Invertebr Neurosci. 14(1):51–58. 10.1007/s10158-013-0161-1 23958799

[evy144-B26] Giribet G. 2008 Assembling the lophotrochozoan (=spiralian) tree of life. Philos Trans R Soc B. 363(1496):1513–1522. 10.1098/rstb.2007.2241 PMC2614230 18192183

[evy144-B27] Grabherr MG, et al 2011 Trinity: reconstructing a full-length transcriptome without a genome from RNA-Seq data. Nat Biotechnol. 29(7):644–652. 21572440 10.1038/nbt.1883 PMC3571712

[evy144-B28] Holland PWH, Marlétaz F, Maeso I, Dunwell TL, Paps J. 2017 New genes from old: asymmetric divergence of gene duplicates and the evolution of development. Philos Trans R Soc B. 372(1713):20150480. 10.1098/rstb.2015.0480 PMC5182412 27994121

[evy144-B29] Hrycaj SM, Wellik DM. 2016 Hox genes and evolution. F1000Research 5:859. 10.12688/f1000research.7663.1 PMC4863668 27239281

[evy144-B30] Hui JHL. 2008. The evolution of clustered homeobox genes (D.Phil.). Oxford: University of Oxford.

[evy144-B31] Hui JHL, et al 2012 Extensive chordate and annelid macrosynteny reveals ancestral homeobox gene organization. Mol Biol Evol. 29(1):157–165. 21727239 10.1093/molbev/msr175

[evy144-B32] Hui JHL, et al 2009 Features of the ancestral bilaterian inferred from *Platynereis dumerilii* ParaHox genes. BMC Biol. 7(1):43. 19627570 10.1186/1741-7007-7-43 PMC2723086

[evy144-B33] Irvine SQ, Martindale MQ. 2000 Expression patterns of anterior Hox genes in the polychaete *Chaetopterus*: correlation with morphological boundaries. Dev Biol. 217(2):333–351. 10625558 10.1006/dbio.1999.9541

[evy144-B34] Katoh K, Standley DM. 2013 MAFFT multiple sequence alignment software version 7: improvements in performance and usability. Mol Biol Evol. 30(4):772–780. 23329690 10.1093/molbev/mst010 PMC3603318

[evy144-B35] Keane TM, Creevey CJ, Pentony MM, Naughton TJ, Mclnerney JO. 2006 Assessment of methods for amino acid matrix selection and their use on empirical data shows that ad hoc assumptions for choice of matrix are not justified. BMC Evol Biol. 6:29. 16563161 10.1186/1471-2148-6-29 PMC1435933

[evy144-B36] Kenny NJ, Namigai EKO, Marlétaz F, Hui JHL, Shimeld SM. 2015 Draft genome assemblies and predicted microRNA complements of the intertidal lophotrochozoans *Patella vulgata* (Mollusca, Patellogastropoda) and *Spirobranchus* (*Pomatoceros*) *lamarcki* (Annelida, Serpulida). Mar Genomics 24:139–146. 26319627 10.1016/j.margen.2015.07.004

[evy144-B37] Kenny NJ, Shimeld SM. 2012 Additive multiple k-mer transcriptome of the keelworm *Pomatoceros lamarckii* (Annelida; Serpulidae) reveals annelid trochophore transcription factor cassette. Dev Genes Evol. 222(6):325–339. 23053624 10.1007/s00427-012-0416-6

[evy144-B38] Kostyuchenko RP, Kozin VV, Kupriashova EE. 2016 Regeneration and asexual reproduction in annelids: cells, genes, and evolution. Biol Bull. 43(3):185–194.

[evy144-B39] Kourakis MJ, Martindale MQ. 2001 Hox gene duplication and deployment in the annelid leech *Helobdella*. Evol Dev. 3(3):145–153. 11440249 10.1046/j.1525-142x.2001.003003145.x

[evy144-B40] Kulakova MA, Cook CE, Andreeva TF. 2008 ParaHox gene expression in larval and postlarval development of the polychaete *Nereis virens* (Annelida, Lophotrochozoa). BMC Dev Biol. 8:61. 18510732 10.1186/1471-213X-8-61 PMC2440741

[evy144-B41] Kulakova MA, et al 2007 Hox gene expression in larval development of the polychaetes *Nereis virens* and *Platynereis dumerilii* (Annelida, Lophotrochozoa). Dev Genes Evol. 217(1):39–54. 17180685 10.1007/s00427-006-0119-y

[evy144-B42] Kumar S, Stecher G, Peterson D, Tamura K. 2012 MEGA-CC: computing core of molecular evolutionary genetics analysis program for automated and iterative data analysis. Bioinformatics 28(20):2685–2686. 22923298 10.1093/bioinformatics/bts507 PMC3467750

[evy144-B43] Lauri A, et al 2014 Development of the annelid axochord: insights into notochord evolution. Science 345(6202):1365–1368. 25214631 10.1126/science.1253396

[evy144-B44] Licciano M, Murray JM, Watson GJ, Giangrande A. 2012 Morphological comparison of the regeneration process in *Sabella spallanzanii* and *Branchiomma luctuosum* (Annelida, Sabellida). Invertebr Biol. 131(1):40–51.

[evy144-B45] Luo Y-J, et al 2018 Nemertean and phoronid genomes reveal lophotrochozoan evolution and the origin of bilaterian heads. Nat Ecol Evol. 2(1):141–151. 29203924 10.1038/s41559-017-0389-y

[evy144-B46] McDougall C, Korchagina N, Tobin JL, Ferrier DEK. 2011 Annelid *distal-less/Dlx* duplications reveal varied post-duplication fates. BMC Evol Biol. 11(1):241. 21846345 10.1186/1471-2148-11-241 PMC3199776

[evy144-B47] Mehr S, et al 2015 Transcriptome sequencing and annotation of the polychaete *Hermodice carunculata* (Annelida, Amphinomidae). BMC Genomics 16(1):445. 26059236 10.1186/s12864-015-1565-6 PMC4462082

[evy144-B48] Miller MA, Pfeiffer W, Schwartz T. 2010. Creating the CIPRES Science Gateway for inference of large phylogenetic trees. In: 2010 Gateway Computing Environments Workshop (GCE). p. 1–8.

[evy144-B49] Morgan TH. 1901 Regeneration. New York: The Macmillan Company; London: Macmillan and Co., Ltd.

[evy144-B50] Morino Y, Hashimoto N, Wada H. 2017 Expansion of TALE homeobox genes and the evolution of spiralian development. Nat Ecol Evol. 1(12):1942. 29085062 10.1038/s41559-017-0351-z

[evy144-B51] Novikova EL, Bakalenko NI, Nesterenko AY, Kulakova MA. 2013 Expression of Hox genes during regeneration of nereid polychaete *Alitta* (*Nereis*) *virens* (Annelida, Lophotrochozoa). EvoDevo 4(1):14. 23638687 10.1186/2041-9139-4-14 PMC3667000

[evy144-B52] Novikova EL, Bakalenko NI, Nesterenko AY, Kulakova MA. 2016 Hox genes and animal regeneration. Russ J Dev Biol. 47(4):173–180. 30272395

[evy144-B53] Özpolat BD, Bely AE. 2016 Developmental and molecular biology of annelid regeneration: a comparative review of recent studies. Curr Opin Genet Dev. 40:144–153. 27505269 10.1016/j.gde.2016.07.010

[evy144-B54] Paps J, Xu F, Zhang G, Holland PWH. 2015 Reinforcing the egg-timer: recruitment of novel lophotrochozoa homeobox genes to early and late development in the pacific oyster. Genome Biol Evol. 7(3):677–688. 25631164 10.1093/gbe/evv018 PMC5322547

[evy144-B55] Patel RK, Jain M. 2012 NGS QC toolkit: a toolkit for quality control of next generation sequencing data. PLoS One 7(2):e30619. 22312429 10.1371/journal.pone.0030619 PMC3270013

[evy144-B56] Pfeifer K, Dorresteijn AWC, Fröbius AC. 2012 Activation of Hox genes during caudal regeneration of the polychaete annelid *Platynereis dumerilii*. Dev Genes Evol. 222(3):165–179. 22569931 10.1007/s00427-012-0402-z

[evy144-B57] Raible F, et al 2005 Vertebrate-type intron-rich genes in the marine annelid *Platynereis dumerilii*. Science 310(5752):1325–1326. 16311335 10.1126/science.1119089

[evy144-B58] Rambaut A. 2007 FigTree. http://tree.bio.ed.ac.uk/software/figtree/, last accessed July 16, 2018.

[evy144-B59] Roensch K, Tazaki A, Chara O, Tanaka EM. 2013 Progressive specification rather than intercalation of segments during limb regeneration. Science 342(6164):1375–1379. 24337297 10.1126/science.1241796

[evy144-B60] Ronquist F, Huelsenbeck JP. 2003 MrBayes 3: Bayesian phylogenetic inference under mixed models. Bioinformatics 19(12):1572–1574. 12912839 10.1093/bioinformatics/btg180

[evy144-B61] Simakov O, et al 2013 Insights into bilaterian evolution from three spiralian genomes. Nature 493(7433):526. 23254933 10.1038/nature11696 PMC4085046

[evy144-B62] Somorjai IML, Somorjai RL, Garcia-Fernàndez J, Escrivà H. 2012 Vertebrate-like regeneration in the invertebrate chordate amphioxus. Proc Natl Acad Sci U S A. 109(2):517–522. 22203957 10.1073/pnas.1100045109 PMC3258630

[evy144-B63] Stierwald M, Yanze N, Bamert RP, Kammermeier L, Schmid V. 2004 The Sine oculis/Six class family of homeobox genes in jellyfish with and without eyes: development and eye regeneration. Dev Biol. 274(1):70–81. 15355789 10.1016/j.ydbio.2004.06.018

[evy144-B64] Sunderland ME. 2010 Regeneration: Thomas Hunt Morgan’s window into development. J Hist Biol. 43(2):325–361. 20665231 10.1007/s10739-009-9203-2

[evy144-B65] Szabó R. 2015 Regeneration and calcification in the *Spirobranchus lamarcki* operculum: development and comparative genetics of a novel appendage (Ph.D.). St Andrews: University of St Andrews.

[evy144-B66] Szabó R, Ferrier DEK. 2014 Cell proliferation dynamics in regeneration of the operculum head appendage in the annelid *Pomatoceros lamarckii*. J Exp Zool B 322(5):257–268. 10.1002/jez.b.22572 24799350

[evy144-B67] Takatori N, et al 2008 Comprehensive survey and classification of homeobox genes in the genome of amphioxus, *Branchiostoma floridae*. Dev Genes Evol. 218(11–12):579–590. 18797923 10.1007/s00427-008-0245-9

[evy144-B68] Tiozzo S, Copley RR. 2015 Reconsidering regeneration in metazoans: an evo-devo approach. Front Ecol Evol. 3:

[evy144-B69] Tomer R, Denes AS, Tessmar-Raible K, Arendt D. 2010 Profiling by image registration reveals common origin of annelid mushroom bodies and vertebrate pallium. Cell 142(5):800–809. 20813265 10.1016/j.cell.2010.07.043

[evy144-B70] Wang KC, Helms JA, Chang HY. 2009 Regeneration, repair and remembering identity: the three Rs of Hox gene expression. Trends Cell Biol. 19(6):268–275. 19428253 10.1016/j.tcb.2009.03.007 PMC4099061

[evy144-B71] Weigert A, et al 2014 Illuminating the base of the annelid tree using transcriptomics. Mol Biol Evol. 31(6):1391–1401. 24567512 10.1093/molbev/msu080

[evy144-B72] Zattara EE, Bely AE. 2011 Evolution of a novel developmental trajectory: fission is distinct from regeneration in the annelid *Pristina leidyi*. Evol Dev. 13(1):80–95. 21210945 10.1111/j.1525-142X.2010.00458.x

[evy144-B73] Zhong Y, Holland PWH. 2011 HomeoDB2: functional expansion of a comparative homeobox gene database for evolutionary developmental biology. Evol Dev. 13(6):567–568. 23016940 10.1111/j.1525-142X.2011.00513.x PMC3399086

[evy144-B74] Zhong Y, Holland PWH. 2011 The dynamics of vertebrate homeobox gene evolution: gain and loss of genes in mouse and human lineages. BMC Evol Biol. 11:169. 21679462 10.1186/1471-2148-11-169 PMC3141429

[evy144-B75] Zwarycz AS, Nossa CW, Putnam NH, Ryan JF. 2016 Timing and scope of genomic expansion within Annelida: evidence from homeoboxes in the genome of the earthworm *Eisenia fetida*. Genome Biol Evol. 8(1):271–281. 10.1093/gbe/evv243 PMC4758240 26659921

